# Etched Fiber Bragg Grating Biosensor Functionalized with Aptamers for Detection of Thrombin

**DOI:** 10.3390/s18124298

**Published:** 2018-12-06

**Authors:** Aliya Bekmurzayeva, Kanat Dukenbayev, Madina Shaimerdenova, Ildar Bekniyazov, Takhmina Ayupova, Marzhan Sypabekova, Carlo Molardi, Daniele Tosi

**Affiliations:** 1 Laboratory of Biosensors and Bioinstruments, National Laboratory Astana, Nazarbayev University, Astana 010000, Kazakhstan; madina.shaimerdenova@nu.edu.kz (M.S.); ibekniyazov@nu.edu.kz (I.B.); takhmina.ayupova@nu.edu.kz (T.A.); msypabekova@nu.edu.kz (M.S.); daniele.tosi@nu.edu.kz (D.T.); 2 School of Engineering, Nazarbayev University, Astana 010000, Kazakhstan; kdukenbayev@nu.edu.kz (K.D.); carlo.molardi@nu.edu.kz (C.M.)

**Keywords:** Optical fiber biosensors, fiber Bragg gratings (FBG), refractive index sensor, aptamer, thrombin, biosensor

## Abstract

A biosensor based on an etched Fiber Bragg Grating (EFBG) for thrombin detection is reported. The sensing system is based on a Fiber Bragg Grating (FBG) with a Bragg wavelength of 1550 nm, wet-etched in hydrofluoric acid (HF) for ~27 min, to achieve sensitivity to a refractive index (RI) of 17.4 nm/RIU (refractive index unit). Subsequently, in order to perform a selective detection of thrombin, the EFBG has been functionalized with silane-coupling agent 3-(aminopropyl)triethoxysilane (APTES) and a cross-linker, glutaraldehyde, for the immobilization of thrombin-binding aptamer. The biosensor has been validated for thrombin detection in concentrations ranging from 10 nM to 80 nM. The proposed sensor presents advantages with respect to other sensor configurations, based on plasmonic resonant tilted FBG or Long Period Grating (LPG), for thrombin detection. Firstly, fabricating an EFBG only requires chemical etching. Moreover, the functionalization method used in this study (silanization) allows the avoidance of complicated and expensive fabrications, such as thin film sputtering or chemical vapor deposition. Due to their characteristics, EFBG sensors are easier to multiplex and can be used in vivo. This opens new possibilities for the detection of thrombin in clinical settings.

## 1. Introduction

Thrombin has an important role in normal and pathological coagulation, with a normal concentration range from nanomolar to low micromolar during blood coagulation [1]. Thrombin levels can be elevated during extra- and intravascular activation of blood coagulation by tumor cells [2]. Thrombin is also involved in diseases such as atherosclerosis, thromboembolic disease, cancer, and inflammatory disease [3,4], and detecting this protein in blood is important for both research and clinical applications [5]. Due to its clinical importance, there is a wide range of work aimed at detecting thrombin and most of it is based on aptamers [1]. 

Aptamers are single stranded oligonucleotides (DNA or RNA) or peptides which are selected in vitro to specifically bind a target [6,7,8]. Bock et al. [9] selected aptamers against human α-thrombin, from which one aptamer, with a dissociation constant K_d_ of 100 nM, was widely used afterwards. Tasset et al. [10] developed a 29-m aptamer against thrombin, with a K_d_ shown to be 200 times lower than 15-m (0.5 nM). Both the selected aptamers formed a G-quadruplex with the target; but, while the shorter aptamer binds the heparin binding site of thrombin, 29-m binds the fibrinogen-binding site [11]. Various thrombin biosensors using these aptamers have been developed and include biosensors based on chemiluminescence [12], electrochemiluminescence [13], electrochemistry [14], Surface Plasmon Resonance (SPR) [15], and optical fiber gratings [16]. 

Optical fiber grating-based sensors have advantages such as a high sensitivity, possibility for multiplexing, small size, light weight, multi-modal sensing capability, immunity to electromagnetic interference, and low fabrication cost [17,18,19]. Several types of optical fiber grating-based biosensors are known to rely on different principles of operation: LPG, EFBG, tilted FBG, microstructured FBG, Photonic Crystal Fibers (PCF) (sometimes regarded as a subtype of microstructured FBG), LPG inscribed in PCF, LPG, and tilted FBG combined with SPR [20,21]. Optical grating sensors such as LPG, EFBG, and tilted FBG sensors have an increasing role in developing chemo- and biosensors due to their label-free nature of measuring RI [21]. Some of these configurations of FBG biosensors have been investigated for developing thrombin biosensors [15,22,23,24]. 

In the biosensor developed by Shevchenko et al. [15], a tilted FBG was coated with a 50 nm gold layer. The thiolated, 15-m Thrombin-Binding Aptamer (TBA) was immobilized by absorption on the gold surface. The sensor was able to distinguish thrombin from other protein solutions used as controls (bovine serum albumin, pepsin, human serum). A modification of this method is the work written by Han et al. [22], where the tilted FBG was subsequently coated with gold, a self-assembled monolayer of 11-mercaptoundecanoic acid, and thrombin aptamers. After the addition of thrombin, a second TBA, attached to a gold nanoparticle, was used in a sandwich assay format. As a result, the transmission intensity change increased compared to a single aptamer format, implying a potential improvement in the sensitivity of thrombin detection. This biosensor had a minimal cross-sensitivity to the outside temperature and the K_d_ values obtained on the biosensor were in good agreement with those stated in the literature. Another thrombin biosensor was developed by Alberts [25], who combined the SPR effect with a tilted FBG-based approach in one sensor, which was able to detect small shifts due to analyte binding with a high accuracy. 

Coelho et al. [23] compared LPG and SPR on optical fibers for thrombin detection (10 to 100 nM). The plasmonic biosensor had a higher sensitivity compared to the LPG biosensor, probably due to its higher sensitivity to RI and insensitivity to thermal variations. An improvement to fiber functionalization procedures was studied by Arghir et al. [24]. They studied the immobilization of (3-mercaptopropyl)trimethoxysilane (to provide thiol groups) on a fiber core, prior to gold coating, and the possible increase of gold adhesion and overall SPR-based fiber optic sensor stability in detecting thrombin with its aptamer. Silanized fibers were shown to be far superior to reference fibers (without the silanization step) in mechanical and chemical stability and reusability, while also promoting gold adhesion during sputtering. This functionalization approach could be used to increase the stability and reproducibility of fiber optic biosensors. 

Cladding-etched FBG or EFBG has recently been investigated to measure RI variation [26]. The interaction between an analyte of interest and the ligand (which is immobilized on EFBG) changes the RI on the surface and thus the Bragg wavelength is shifted and the grating reflectivity is changed. In this way, the concentration of the analyte can be measured by analyzing FBG spectral changes (in transmission or reflection) [27]. The optical fabrication of an EFBG is straightforward and fast, due to the availability of chemical etching fluids such as hydrofluoric acid, which can remove the cladding in a few minutes at a controlled rate. Alternatives, such as core-exposed micromachined gratings [28], require the availability of a laser micromachining station, and are therefore harder to manufacture. 

Although EFBG sensors are less sensitive than tilted FBG, LPG, or SPR-assisted tilted FBG and LPG, they also offer other advantages over these sensing mechanisms. EFBG sensors also include a simple tunability of their sensitivity [29]. While tilted FBG and LPG work in transmission, EFBG measures reflection and does not need a polarization control [30]. To be able to work in reflection, a broadband mirror has to be fabricated in the tip of the cleaved fiber (often as a thin film), which requires an additional fabrication step [25]. For instance, LPG sensors have to be precisely cut after the grating to avoid the formation of interference fringes (inside the attenuation bands) and the tip is then coated with a reflecting layer [31]. Compared to LPG, where light from a guided mode is coupled to a forward propagating mode producing multiple attenuation bands in transmission [32], EFBG with its single or few core modes offers a simple output signal for spectral peak detection [26]. Multiplexing capabilities of LPG are limited due to its multiple resonance peaks; its measurement accuracy is also limited because of its broad line-width at full-width at half maximum [33]. While ways of improving the adhesion of the gold layer for plasmonic optical fiber sensors are being investigated [24], special equipment to deposit gold is still required to develop these types of biosensors [24] or the use of gold nanoparticles is needed [34]. Alternatively, the glass surface of EFBG can be silanized with different silane-coupling agents for further immobilization of bioreceptors, without the need for the deposition of a gold layer. Due to the abovementioned advantages of EFBG sensors, we aimed at developing an EFBG biosensor for the detection of thrombin, which is a clinically important biomarker in many diseases. In this study, we report an EFBG-based biosensor based on a grating operating in the third optical window, selecting the thrombin binding aptamer as a ligand for the detection of thrombin in different concentrations.

## 2. Materials and Methods

### 2.1. Materials

Single-mode pure silica fibers (SMF-28C, 10/125 μm core/cladding diameter) with inscribed Bragg gratings (1.0 cm length, >90% reflectivity, ~1550 nm central wavelength) were purchased from Technica Optical Components (Atlanta, GA, USA) Oleic acid, 3-Aminopropyl)triethoxysilane (APTES), glutaraldehyde, phosphate buffered saline (PBS), thrombin protein, and sulfuric acid were purchased from Sigma Aldrich (Darmstadt, Germany). Sucrose, acetone, sodium dodecyl sulfate (SDS), bovine serum albumin, Tris-HCl, KCl, Na_2_HPO_4_, MgCl_2_, and NaCl were obtained from Thermo Fisher Scientific (Runcorn, UK). Amine-modified thrombin binding aptamer (TBA) (5′-AmC6FTTTTT-AGTCCGTGGTAGGGCAGGTTGGGGTGACT-3′) was synthesized by Sigma Aldrich.

Buffers used in this work:

- Affinity buffer [10] (Sensor-aptamer + thrombin): 50 mM Tris-HCl pH 7.4, 250 mM NaCl, 5 mM MgCl_2_
- Sodium citrate buffer (for resuspension of stock thrombin protein): 100 mM sodium citrate, 100 mM citric acid
- Measurement buffer [23] (for regeneration): 10 mM Tris-HCl pH 7.4, 100 mM KCl

### 2.2. Setup

**Figure 1:**
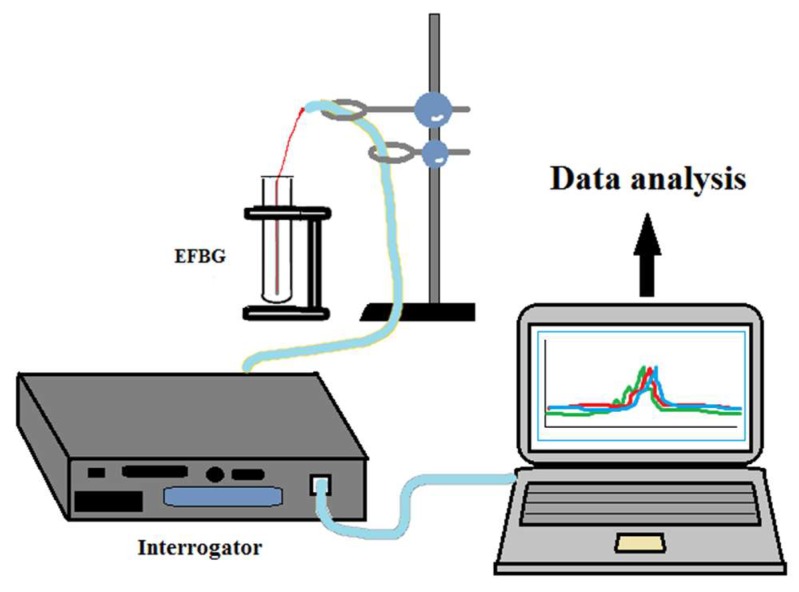
Setup used for developing a thrombin biosensor using an optical sensing interrogator and EFBG attached to a plastic stick.

The setup used for the development and interrogation of the thrombin biosensor is shown in Figure 1. The peak wavelength shift and the reflection spectra of FBG were measured using an si255 optical sensing interrogator (Micron Optics Inc., Atlanta, GA, USA). It has a resolution (also known as pixel size) of 8 pm over a 160 nm bandwidth (20,000 points for wavelength grid). It is able to distinguish an area of 8 pm between two detected signals. The interrogator has eight physically separated channels. The interrogator system contains a wide-range swept wavelength laser as a light source and a scan range of 1460–1620 nm. The interrogator has been used for the detection of the EFBG spectra throughout each fabrication and functionalization step, as well as for real-time detection during thrombin measurement. The instrument performs the spectral measurement of the reflection spectra of each grating; the Bragg wavelength is estimated from the EFBG spectra using the centroid demodulation of the spectrum (a signal processing technique), described in [35], which achieves a limit of detection of ~0.2 pm. 

### 2.3. Etching and Calibration

The fiber surrounding the FBG was stripped and the buffer layer in the active sensing area was removed using acetone. Then, the sensor was attached to a plastic stick (diameter about 4 cm; length 20 cm) to ensure that the etching occurred in the tip. Etching was done in HF solution (48% concentration) topped with oleic acid (in order to protect the etched fiber and the environment [36]) in a fume hood (Waldner Secuflow airflow controller; Waldner Inc., Boston, MA, USA) at room temperature (25 °C). Etched FBG sensors were then calibrated in solutions of sucrose in water (1.5%; 3.1%; 6.2%; 12.5%; 25.0% and 50.0% *w/v*). In our studies, we used calibrated solutions of sucrose with estimated refractive indices according to [37]. In total, several FBG sensors were etched and calibrated. 

### 2.4. Silanization of FBG Sensors

**Figure 2:**
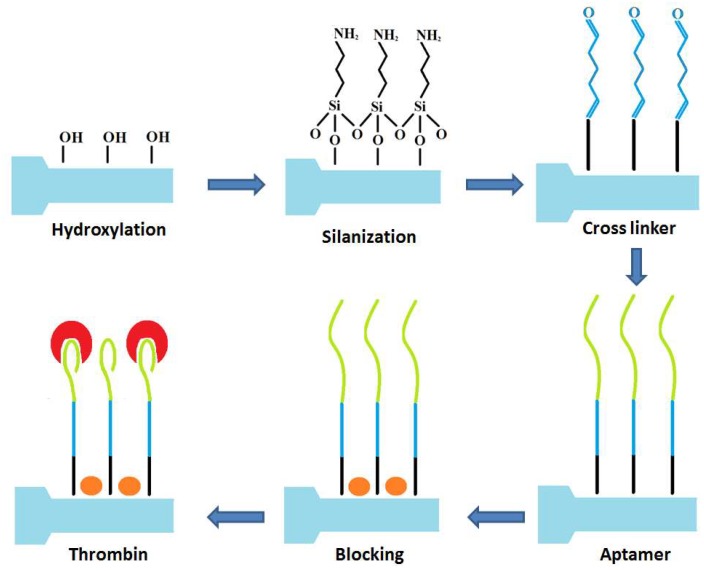
Schematic representation of developing a biosensor based on etched FBG for thrombin detection.

The process for functionalization of the EFBG into a thrombin biosensor is sketched in Figure 2. The etched FBG was treated with Piranha solution (3:1 *v/v* H_2_SO_4_:H_2_O_2_) to clean the surface and make it more hydrophilic in nature for further functionalization. It was further silanized with 1% APTES in ethanol (absolute) for 30 min and rinsed with ethanol. The sensors were then heat treated at 110 °C for 30 min and incubated in 25% glutaraldehyde in PBS for 1 h [38] and washed in water [39]. Following this, the sensor was treated with aminated thrombin aptamers for 4 h. Unbound aptamers were washed away with 0.2% sodium dodecyl sulfate in PBS. To reduce non-specific binding, the surface was blocked with 1% bovine serum albumin (in PBS) for 30 min. The thrombin protein in different concentrations (10, 20, 40, 80 nM in affinity buffer) was incubated with sensors for 2 h in each test. 

### 2.5. Atomic Force Microscopy Analysis of the Functionalized Surface

High-resolution topographical characterization of the surfaces was carried out using Atomic Force Microscopy (AFM) SmartSPM 1000 (AIST-NT Inc., Novato, CA, USA) in AC-Mode (non-contact mode) of AFM scanning. All the AFM measurements were performed with a scan range of 1500 nm in X-Y and height Z was set automatically, with a scanning rate 0.2 Hz. AFM images were obtained using a super sharp type “NSG30_SS” cantilever, with a tip radius curvature of up to 5 nm, force constant of 22–100 N/m, and resonance frequency of 200–440 kHz in air (Tips Nano).

## 3. Results

**Figure 3:**
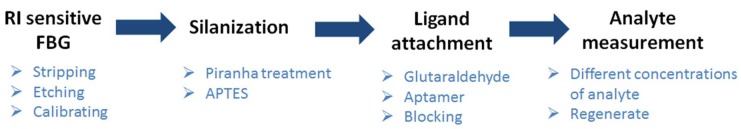
Overview of developing a biosensor based on etched FBG.

The EFBG biosensor was characterized according to the schematic shown in Figure 3. The etching process was performed by immersing the FBG in a solution of HF at 48%, maintained at room temperature in a fume hood. The biosensor was fabricated using this etching process, applied over ~27 min. The cladding diameter was reduced progressively during etching, where the sensitivity started to appear.

**Figure 4:**
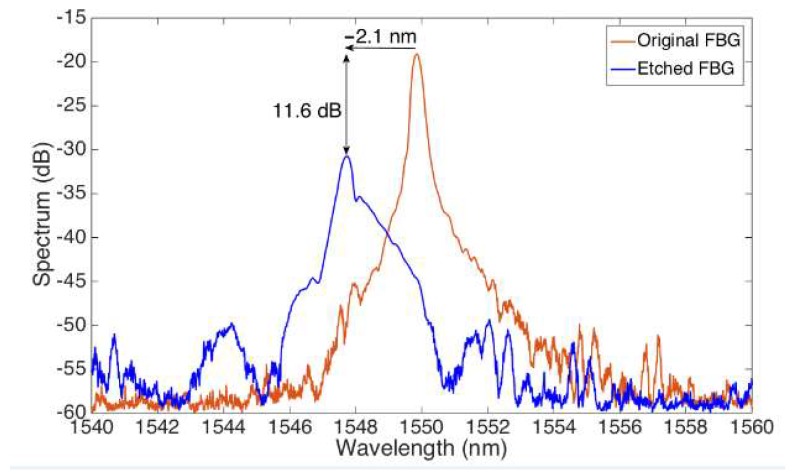
Spectrum of the original FBG prior etching, and of the EFBG obtained by etching the FBG for ~27 min. The chart reports the spectra observed on the FBG interrogator.

Figure 4 shows the change in the optical spectrum from the original FBG, after the grating has been etched to reach a cladding thickness of approximately 25 μm in diameter. The original FBG has a Bragg wavelength of 1549.98 nm. During the etching process, we observe both a change of wavelength of −2.1 nm, and a reduction of the FBG peak of 11.6 dB, which correspond to the evanescent wave effect of the etched sensor, resulting in this extra attenuation on both the forward and backward waves. 

**Figure 5:**
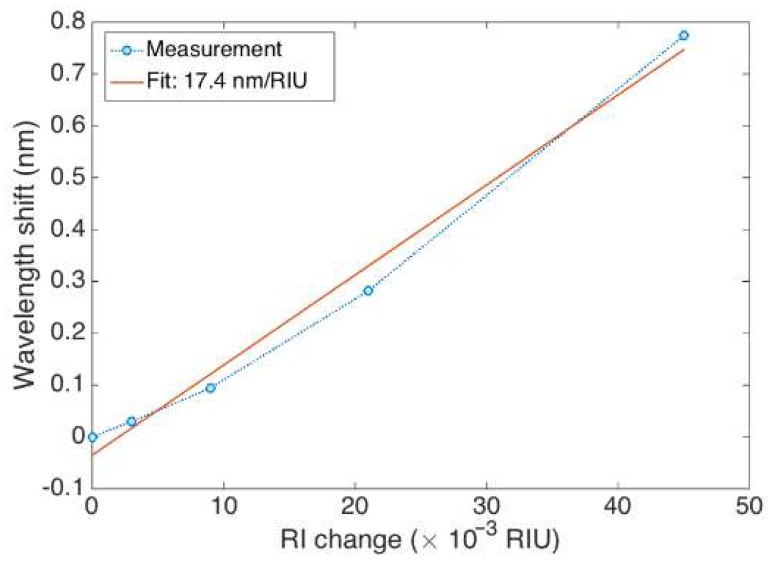
RI calibration of the EFBG, showing the calibration data for different RI values of sucrose in water (1.5%; 3.1%; 6.2%; 12.5%; 25.0%; and 50.0% *w/v*) and the corresponding wavelength shift (1.5% set at 0). Linear regression is used to estimate the sensitivity (17.4 nm/RIU).

The FBG hereby fabricated has been calibrated for determining the RI sensitivity, using a method previously used in [30] that consists of immersing the grating in solutions of different RI values formed by water/sucrose mixtures (1.6%, 3.1%, 6.3%, 12.5%, 25.0%); the refractive index change is ~1.85 × 10^−3^ RIU for each 1% of sucrose, for such values of the refractive index. The wavelength shifts towards the longest values when the RI increases, with an estimated sensitivity of 17.4 nm/RIU and a linearity coefficient of 0.993 is obtained, as seen in Figure 5.

**Figure 6:**
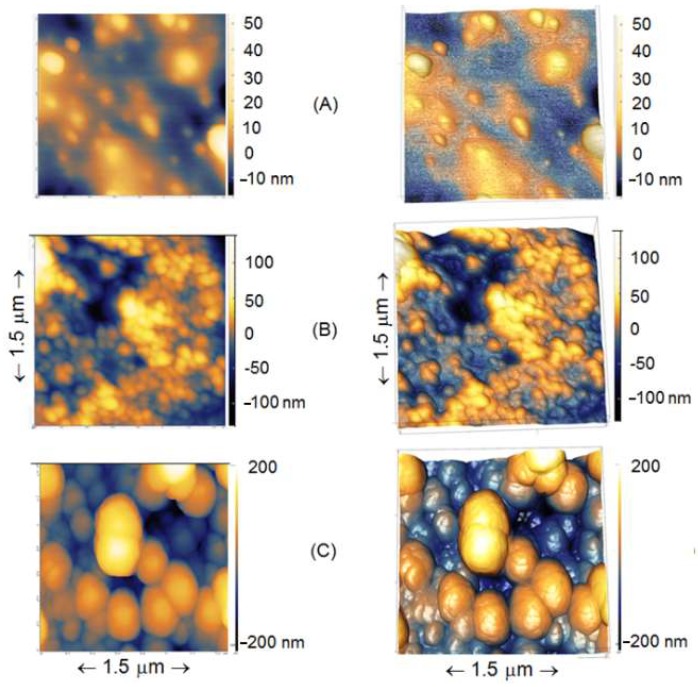
High-resolution 2D (**left column**) and 3D (**right column**) AFM topographical images of etched glass optical fiber surfaces in air. (**A**) Etched surface (not functionalized); (**B**) Etched + APTES; (**C**) Etched + APTES + Glutaraldehyde. Scanning sizes for three images are XY 1.5 µm × 1.5 µm and an average height is in a range −200 to +200 nm.

The surfaces of control samples (treated the same way as sensors) were studied on AFM to confirm surface modification. High-resolution AFM topographical images of control etched glass optical fiber surfaces treated with APTES and glutaraldehyde and without treatments are shown in Figure 6. 

**Figure 7:**
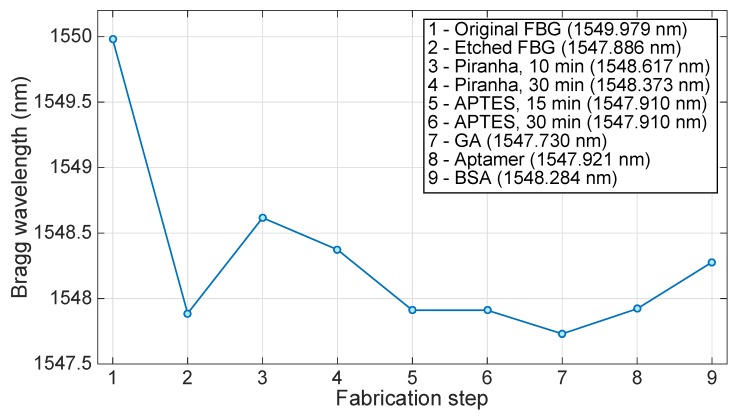
Change of Bragg wavelength through the fabrication and functionalization of the EFBG to thrombin sensing. The chart shows the estimated Bragg wavelength after each fabrication step that precedes the thrombin detection. In the figure, key GA stands for glutaraldehyde, while BSA stands for bovine serum albumin.

**Figure 8:**
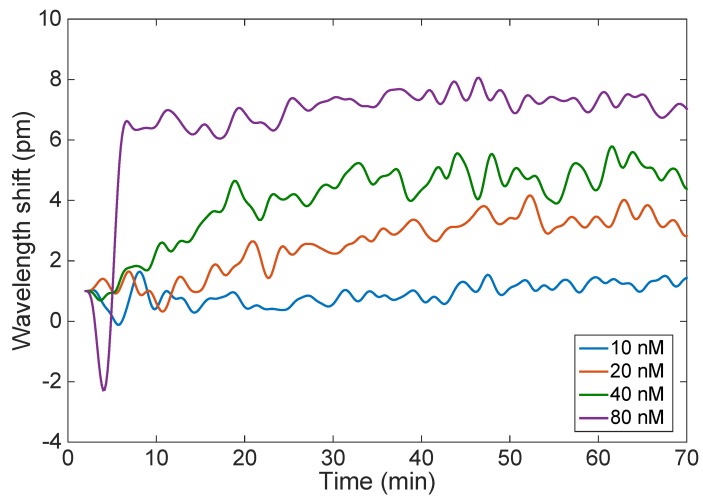
Wavelength shift observed for 70 min detection of thrombin for the EFBG, measured at different concentrations of thrombin ranging from 10 nM to 80 nM.

Figure 7 shows the estimated Bragg wavelength after each fabrication step that precedes the thrombin detection. The sensor performance was tested against varying concentrations of thrombin (10, 20, 40, and 80 nM) in affinity buffer. The main measurement result is shown in Figure 8, which compares the wavelength shift obtained when the EFBG is exposed to different concentrations of thrombin as exposure time progresses. All measurements have been initialized by setting the time to zero at the first moment of exposure, and the wavelength is tracked with a 10 s sampling rate. After each measurement, thrombin is washed out from measurement buffer. We observe that the Bragg wavelength progressively deviates from the initial position by a few picometers, probably due to instrument drift or the binding-rebinding of thrombin on the sensor surface. In the first 10 min, the sensor response has not reached the regime and the wavelength appears to float. After 20 min, the response at different concentrations appears to stabilize, as the Bragg wavelength appears to remain quite stable for the rest of the measurement. Given the urgent time in clinical settings, 20 min can be considered as good enough [14].

**Figure 9:**
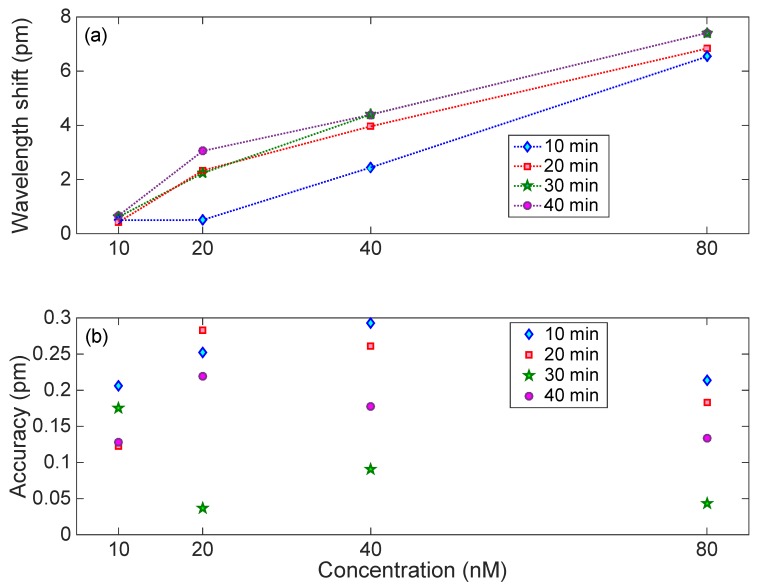
Analysis of the response of the EFBG to thrombin. (**a**) Wavelength shift observed for each concentration, after 10, 20, 30, and 40 min from the start of the experiment. (**b**) Accuracy of the detection, estimated as the standard deviation of the Bragg wavelength recorded over 1 min exposure.

The results of Figure 8 have been further analyzed, to evaluate the sensor response over time and to evaluate the uncertainty. Bragg wavelength shift (recorded over 1 min) is plotted against thrombin concentrations (Figure 9a). At the lowest measured concentration of thrombin (10 nM), it is difficult to distinguish how the wavelength shift changes with time; this is better for higher concentrations of thrombin. Accuracy was estimated as the standard deviation of the Bragg wavelength change recorded over one minute at constant thrombin concentrations (Figure 9b). It can be clearly seen that as time passes, the accuracy of the measurement is improved. For most measurements, the Bragg wavelength deviation stays within 0.05 to 0.3 pm, which is the same value estimated for the Bragg wavelength detection on the interrogator (0.2 pm). 

## 4. Discussion

To make FBG sensitive to surrounding RI, its cladding has to be depleted to ensure the interaction of the evanescent wave field of the propagating mode with the surrounding medium or analyte [19]. Chemical etching using HF is an attractive method of choice because of its simplicity and it is widely used [36]. As reported in [40], HF etching is a fast method that can etch a diaphragm in a few minutes, operating at a progressive rate. During the etching process, the spectrum is monitored in order to track the wavelength shift due to the change of sensitivity of the EFBG to the external refractive index. Our results suggest an etching rate of approximately 3.7 ± 0.5 µm/min, which has been estimated by linearizing the relationship between the fiber diameter (observed under an optical microscope) and time; this estimate is not performed on the reported EFBG, but on other etched gratings that have been used for this task. The reported etching rate of an optical fiber is different, according to the literature review. One study reported an etching rate of 0.65 µm/min at 24 °C for 125 µm fiber using 24% HF solution [41]. Additionally, an etching rate of 1.45 µm/min was observed for 48–52% HF [42], 4.1 µm/min using 49% HF [43], and 3.6 µm/min for 50% HF on a 116 µm fiber [44]. Thus, increasing the concentration of HF in etchant increases the rate of etching. Furthermore, different etching rates could be attributed to the different chemical compositions of the cladding (pure Si) and Ge-doped core [45]. 

A Bragg wavelength shift of 2.1 nm is measured when the fiber diameter is reduced from 125 µm to 25 µm. This shift occurs due to both a local temperature increase of the chemical reaction and a decrease of the cladding thickness [29]. A rapid decrease of the Bragg wavelength was observed after the fiber diameter reached 20 µm because of an increasing interaction between HF and the fundamental guided mode of the fiber [41]. Diameters of etched FBG sensitive to surrounding RI are reported to be different in the literature: 5 µm [18,46,47], 8 µm [41], 10 µm [43], 20 µm [47], and 37.5 µm [42]. As observed in [48], the largest sensitivity values are obtained for a longer etching process, resulting in a thinner cladding of the fiber. However, this process results in an increased evanescent field, and leads to a multi-modality that causes the FBG spectrum to significantly enlarge, deforming from its original shape. The etched spectrum shown in Figure 4 has an inferior RI sensitivity to the thinnest grating reported in [48], but its regular shape and higher signal to noise ratio allow an easy detection with the centroid method (or by the bandwidth-tracking method used by default by the interrogator) [35]. The resulting FBG spectrum is still well-detectable by the FBG interrogator, and the wavelength shift corresponds to the change of sensitivity to outer RI. Moreover, the thicker diameter of the fiber resulting from etching allows a higher mechanical strength. As seen from Figure 5, etching FBG resulted in a sensor sensitive to the SRI, suggesting the possibility to use it as a biosensor.

In biosensor development, one way of attaching a ligand on an optical fiber is silanization, with silane-coupling agents to attach ligands for biosensor development. The immobilization of TBA on an EFBG surface sensitive to refractive index change is achieved using a silane-coupling agent (APTES) and a cross-linking agent (glutaraldehyde). Similarly, an LPG sensor was functionalized with APTES and then with glutaraldehyde for the attachment of a ligand to fabricate a stable label-free biosensor for the detection of bacteria [49]. Prior to functionalization, an optical fiber is pretreated to get a hydroxylated surface. Treatment of EFGB with Piranha resulted in an increase of the wavelength at first and then its slight decrease, with an overall shift of approximately 0.5 nm (Figure 7). Silane-coupling agents are silicon-based materials which have a general formula of R′(CH_2_)_n_Si(OR)_3_, where R′ is an organofunctional group and R is a hydrolysable alkoxy group [50]. Their alkoxy groups are converted to silanol groups (SiOH) when silanes are mixed with water/ethanol solution. They form hydrogen bonds with surface hydroxide groups on metals, while the excess of silanol groups forms a siloxane network (Si-O-Si). This network is chemically stable and shows resistance to corrosion [51]. Additionally, silanization can result in a surface with a greater variety of functional groups, depending on the silane-coupling agent used. Thus, treatment with APTES and 3-(2-aminoethylamino)propyl-trimethoxysilane yields an aminated surface [52,53], glycidyloxypropyl-trimethoxysilane yields an epoxide surface [54], and (3-mercaptopropyl) trimethoxysilane renders the surface with thiol groups [54]. The attachment of APTES on EFBG progresses more rapidly in the beginning and seems to stabilize after 15 min. A final shift of 0.46 nm was observed during its attachment (absolute ethanol as a measurement medium). A Bragg wavelength shift of 28 pm was observed by Saini et al. [18] when immobilizing 1% APTES on FBG. They also reported that silanization occurred in the first 15 min of incubation with APTES. 

AFM analysis of control samples treated similarly to biosensors showed that the surface morphology becomes rougher with surface modification (Figure 6): etched < etched + APTES < etched + APTES + glutaraldehyde, thus confirming successful modification of the studied surfaces. The results are similar to those obtained by Sun et al. on FBG [55], where a gradual increased roughness is also demonstrated. The thickness of APTES layers in different studies was reported to be 0.7 nm (on Silicon wire) [38] or 1.8 nm [56]. In addition, even the AFM cantilevers we used had up to 5 nm tip radius curvature, and accordingly, the high-resolution AFM images obtained demonstrate the fact that for some other new cantilevers from the box, a tip radius curvature in reality was even sharper (i.e., from 2–4 nm). Therefore, for some regions on the image, it is clearly shown that the thickness of APTES, glutaraldehyde in our study, was shown to range from 1–5 nm. Such a difference in the obtained thickness could be due to the number of layers formed on the surface and for some areas, on the etched fiber, there were non-homogenously distributed APTES, with glutaraldehyde particles throughout the fiber surface. It is well-illustrated in Figure 6 that some non-uniformity of deposited biological substances is present.

After the immobilization of aptamers, the performance of the sensor in terms of detecting different concentrations of thrombin was tested. Clinically relevant concentrations of thrombin depend on the disease studied. Thus, for blood coagulation disorders, the thrombin concentration changes in a range from 1 to 500 nM and biosensors able to detect this range would thus be of clinical use [15]. The same concentration of thrombin used in the work by Coelho et al. [23] was used in this work. For all measured concentrations of thrombin, there was a tendency of an increased output signal for functionalized biosensors. Coelho et al. [23] were able to detect 10 nM thrombin with a wavelength shift of 3.5 nm and a resolution of 0.54 nM. Although our biosensor has a lower performance, the EFBG method with silanization-based functionalization used in this study is attractive because it combines the features of fiber optic biosensors with a facile fabrication process. In terms of the working principle, EFBG differs from other grating-based technologies, because it allows the simple measurement of the Bragg wavelength in reflection, a feature that is already integrated in most commercial interrogators and does not require additional manufacturing steps. Tilted FBG and LPG, instead, operate in transmission, which is not practical for a sensing probe because light must be transmitted through the sensing region. It is possible to fabricate a mirror on the tip to detect the transmission reflection spectrum, but this fabrication requires an additional step. The two main alternatives to gratings (tapers and SPR sensors) are also harder to manufacture. SPR requires polarization control and operates in transmission, while tapers are very fragile and the fabrication process yields a low throughput for narrow tapers. It is worth mentioning that the SPR sensors are typically orders of magnitude more sensitive (nm/RIU figure) than the EFBG; however, while SPR is concerned with the detection of a large spectral hole, a single-mode EFBG requires resolving small shifts of a narrow spectrum, which can be implemented using signal processing methods [35]. An accuracy of a 0.1–1 pm can be typically achieved. As demonstrated in the previous figures, this allows detecting at the tens of nM level, which is compatible with many biosensors applications. The reduced sensitivity also has the consequence of lowering the amount of evanescent power dissipated in the surrounding of the biosensor. Another advantage of the EFBG with respect to the other methods is that the EFBG occupies a narrow wavelength spectrum, which allows an efficient use of the bandwidth and the possibility to create sensing networks by stacking multiple sensors in the same fiber. In the future, by using multiple sensing regions, it will be possible to detect different analytes (other biomarkers) or use controls (for temperature compensation or other proteins) on the same fiber. Moreover, working in reflection mode allows EFBG sensors to be more compatible for in vivo sensors than LPG and tilted FBG sensors, which work in transmission.

## 5. Conclusions

We have reported the first fiber optic biosensor functionalized for thrombin detection based on a standard etched fiber Bragg grating. The technology used is simple, reliable, and cost effective due to the fact that a complex optical structure, such as tilted FBG, LPG, or plasmonic-based features, is not involved in our sensor fabrication. At first, the grating is etched in a 48% HF solution, which introduces sensitivity to the external RI; the sensitivity of the EFBG Bragg wavelength with respect to RI is 17.4 nm/RIU. Subsequently, the fiber is functionalized for selective thrombin sensing by means of silanization with APTES and further immobilizing the thrombin binding aptamer. AFM measurements of the modified surfaces (etched → etched + APTES → etched + APTES + glutaraldehyde) showed increased roughness, suggesting successful modification of the surface. Experimental measurements have been carried out, exposing the functionalized EFBG to thrombin in concentrations ranging from 10 nM to 80 nM. We observed a different shift of the Bragg wavelength for each concentration value, reaching a final value of 0.5 pm (10 nM), 2 pm (20 nM), 4 pm (40 nM), and 7 pm (80 nM), with a standard deviation of 0.3 pm. Concentrations used in this study are in the range of clinically important values. Although the sensitivity of EFBG is considered lower than other FBG-based sensors, it has a number of advantages which they do not possess; moreover, it permits the analysis of data. Future work will aim to improve the fabrication process by employing a large batch of sensors and functionalizing other aptamers for use in other biosensing applications, such as for the detection of biomarkers implicated in cancer or infectious diseases.
